# Enzymatically crosslinked gelatin hydrogel promotes the proliferation of adipose tissue-derived stromal cells

**DOI:** 10.7717/peerj.2497

**Published:** 2016-09-27

**Authors:** Gang Yang, Zhenghua Xiao, Xiaomei Ren, Haiyan Long, Hong Qian, Kunlong Ma, Yingqiang Guo

**Affiliations:** 1 Department of Medical Information and Engineering, School of Electrical Engineering and Information, Sichuan University, Chengdu, Sichuan, China; 2 Department of Cardiovascular Surgery, West China Hospital, Sichuan University, Chengdu, Sichuan, China; 3 Center of Engineering-Training, Chengdu Aeronautic Polytechnic, Chengdu, Sichuan, China; 4 Department of Orthopaedics, Yongchuan Hospital, Chongqing Medical University, Yongchuan, Chongqin, China

**Keywords:** Gelatin, Hydrogel, Crosslink, Transglutaminase, Stem cell, Adipose

## Abstract

Gelatin hydrogel crosslinked by microbial transglutaminase (mTG) exhibits excellent performance in cell adhesion, proliferation, and differentiation. We examined the gelation time and gel strength of gelatin/mTG hydrogels in various proportions to investigate their physical properties and tested their degradation performances *in vitro*. Cell morphology and viability of adipose tissue-derived stromal cells (ADSCs) cultured on the 2D gel surface or in 3D hydrogel encapsulation were evaluated by immunofluorescence staining. Cell proliferation was tested via Alamar Blue assay. To investigate the hydrogel effect on cell differentiation, the cardiac-specific gene expression levelsof Nkx2.5, Myh6, Gja1, and Mef2c in encapsulated ADSCs with or without cardiac induction medium were detected by real-time RT-PCR. Cell release from the encapsulated status and cell migration in a 3D hydrogel model were assessed *in vitro*. Results show that the gelatin/mTG hydrogels are not cytotoxic and that their mechanical properties are adjustable. Hydrogel degradation is related to gel concentration and the resident cells. Cell growth morphology and proliferative capability in both 2D and 3D cultures were mainly affected by gel concentration. PCR result shows that hydrogel modulus together with induction medium affects the cardiac differentiation of ADSCs. The cell migration experiment and subcutaneous implantation show that the hydrogels are suitable for cell delivery.

## Introduction

In the field of tissue engineering and regenerative medicine, researchers have been searching for ideal biomaterials that mimic the structure and composition of the extracellular matrix (ECM) and can be used for cell 3D culture and cell transplantation *in vivo*. Hydrogel materials are one of the most important areas of research in biological materials because of their high moisture content and high plasticity (Johnson & Christman, 2013; Radhakrishnan, Krishnan & Sethuraman, 2014; Toh & Loh, 2014). Numerous kinds of hydrogel materials have emerged, including natural materials, such as collagen, gelatin, hyaluronic acid, laminin, chitosan, and sodium alginate, and synthetic materials, such as polylactide, polylactide-co-glycolic acid copolymer, polyethylene glycol, polycaprolactone, and polyacrylamide (El-Sherbiny & Yacoub, 2013; Gibbs et al., 2016). Gelatin, a well-known biological material because of its good biocompatibility, is the degraded product of collagen, which is the main component of native ECM. Compared with that of collagen, gelatin manufacturing process is relatively simple, and its market price is cheaper than the former. However, the natural gelatin hydrogels are highly hydrated and possess poor mechanical stability and durability, which critically limit their wide-spread application; thus, the manufacture of gelatin-based biomaterials frequently requires a certain degree of crosslinking for stabilizing gelatin macromolecules. Several approaches for crosslinking gelatin, such as physical crosslinking, chemical crosslinking, and enzymatic crosslinking, were used.

In conventional physical crosslinking of gelatin, an aqueous solution of several percent gelatin turns to a transparent elastic hydrogel upon cooling below ∼35 °C, and crosslinking occurs via random coil gelatin molecules turning to the ordered triple helix conformation of collagen. However, the thermoreversibility of the hydrogel makes it melt at physiological temperature. Other physical crosslinking methods, such as plasma treatment, often result in low crosslinking extent of gelatin macromolecules because crosslinking occurs only at the surface of the material (Ratanavaraporn et al., 2010). Numerous researchers have aimed to build more stable hydrogels by using UV light or chemical crosslinkers. Photocrosslinked hydrogels usually present short gelation time and are chemically stable and mechanically strong, but both photo-initiators and UV light required for the photopolymerization reaction may lead to cell death (Jin et al., 2010). Chemical crosslinking of gelatin refers to the use of chemical reagents, such as glutaraldehyde, formaldehyde, 1-(3-dimethylaminopropyl)-3-ethyl-carbodimide hydrochloride, and genipin, as a chemical crosslinker (Tseng et al., 2013). Despite the improved mechanical strength and proteolytic stability of crosslinked gelatin hydrogels, chemical crosslinkers often elicit either cytotoxic side-effects or immunological responses from the host (Yung et al., 2007; Li & Liu, 2009).

Enzymatic crosslinking is a new approach to biomaterial crosslinking. The enzymes, including tyrosinase and transglutaminase, are currently known as crosslinkers for numerous kinds of proteins, including gelatin and collagen (Yung et al., 2007; Spurlin et al., 2009; Kuwahara et al., 2010; Taddei et al., 2013). Transglutaminase is highlighted as the best studied enzyme system involved in protein-based hydrogel crosslinking for tissue engineering approaches, because it can offer intimate integration between the *in situ* formed hydrogel and the native host tissue (Teixeira et al., 2012). Moreover, the hydrogel catalyzed by transglutaminase is mechanically stronger and more stable than that catalyzed by tyrosinase (Chen et al., 2003). However, owing to the relatively high price of these enzymes compared with chemical crosslinkers, the enzymatic crosslinking method for gelatin had been rarely used until microbial transglutaminase (mTG) was discovered. mTG, which is derived from streptomycetes, exhibits high specific activity over a wide range of temperature and pH and is Ca^2+^ independent. mTG has been extensively utilized in the food industry, enhancing the functional properties of protein-rich food through covalent crosslinking (Halloran et al., 2008; Wangtueai, Noomhorm & Regenstein, 2010).

At present, few studies have reported on gelatin hydrogel crosslinked by mTG as a cell scaffold material (Paguirigan & Beebe, 2007; Yung et al., 2007; Kuwahara et al., 2010; Bode et al., 2011; De Colli et al., 2012; Bode et al., 2013; Da Silva et al., 2014). Numerous issues remain worth studying. For example, we know that transglutaminase is non-toxic and exerts no side-effects on several cell types, but we do not know its effects on other cell types, such as adipose tissue-derived stromal cells (ADSCs). ADSCs are a kind of adult stem cells with rich cell sources and can be obtained by minimally invasive surgery, such as subcutaneous liposuction. ADSCs present multiple differentiation potential and can differentiate into osteoblasts, chondrocytes, adipocytes, and cardiomyocytes (Wilson, Butler & Seifalian, 2011; Wankhade et al., 2016). Therefore, ADSCs present a considerable potential source of stem cells for tissue engineering research and clinical applications (Pikula et al., 2013; Suzuki et al., 2015; Naderi et al., 2016; Pak et al., 2016).

How will the degradation of gelatin/mTG hydrogels be affected by cell secretion after ADSCs are inoculated on the hydrogels? On the other hand, how does the degradation of materials affect cell growth? Extremely little knowledge on these topics is available. In this study, we will analyze the degradation of gelatin/mTG hydrogels *in vitro* and *in vivo* with or without cell inoculation and evaluate cell growth in 2D or 3D culture to determine whether the material is suitable as a cell scaffold.

At present, whether gelatin/mTG hydrogel can be used as a cell carrier for *in vivo* transplantation after inoculated with ADSCs and whether the release of cells from the hydrogel is controllable remain unclear. If the cells are released too quickly, rapid cell loss from the implantation site will occur, thereby undermining the purpose of tissue repair and regeneration. In addition, whether the material is conducive to cell migration is unclear. Cell migration often facilitates the organization of the capillary network surrounding the implanted hydrogel to establish blood supply. In this study, we will design an *in vitro* 3D model to simulate cell migration inside the hydrogel with the aim of providing evidence for *in vivo* animal experiments in the future.

## Materials and Methods

### Preparation of gelatin hydrogels

Gelatin gel formation was initiated by mTG addition. For hydrogel preparation, gelatin powder (type A, 300 Bloom; Sigma–Aldrich, MO, USA) was weighed and dissolved in phosphate-buffered saline (PBS) at 50 °C and then sterilized as rapidly as possible through 0.22 µm filters to prevent filter blockage by the cooling gel. The mTG (Bomei, China, enzyme activity units > 100 U per gram) solution was prepared by dissolving mTG in PBS to obtain 10% (wt, weight ratio) solution and then sterilizing through 0.22 µm filters. Gelatin/mTG hydrogels were prepared by mixing a certain amount of 10% mTG solution with different concentration of gelatin solutions according to the experimental need. To determine the effects of different gelatin concentration (1%, 2%, 4%, 6%, 8%, and 10% (w/v, weight/ volume)) on the gelation time and gel strength of resultant hydrogels, and the mTG dosage was retained at 10 U/g pro (enzyme activity units per gram of protein). Here, the protein is gelatin. To determine the effects of mTG dosage (2, 5, 10, 20, and 40 U/g pro) on gelation time and gel strength of the resultant hydrogels, and the concentration of gelatin solution was maintained at 4% (w/v).

### Gelation time and gel strength test

For gelation time test, 2 ml of gelatin/mTG solution was added in a transparent glass vial and incubated at 37 °C, and the onset of gelling detected through the vial inverting method was recorded as gelation time. Six repeated measurements for each type of gelatin/mTG hydrogels were performed. For the gel strength test, 6 ml of gelatin/mTG mixing solution were added into a 35 mm culture dish and incubated at 37 °C for 2 h; then, the dish was fixed on the platform of a mechanical testing apparatus (HPB, Handpi, China). The detecting probe was a flat-head stainless-steel cylinder (12.5 mm in diameter, 10 mm in height) attached to a digital pull-and-push dynamometer (HP-20 Handpi, China). The probe was loaded down onto the hydrogel at a constant rate of 1.0 mm/s at room temperature (∼24 °C). The advance was stopped when the probe front reached to a 5 mm depth from the gel surface. Meanwhile, the value of loading force was recorded automatically by a mechanical measurement software (Yueqing Handpi Instruments Co., Ltd, Zhejiang, China). The peak value of the recorded curve was acquired and converted to gel strength. Six repeated measurements for each type of gelatin/mTG hydrogels were performed.

### Hydrogel degradation test

The degradation of hydrogel materials in two groups was tested. The first group consisted of cell-free hydrogels, and the second group consisted of cell-containing hydrogels. To prepare cell-free hydrogels, twelve 35 mm culture dishes were pre-weighed with an electronic balance and respectively marked. Subsequently, 2%–8% concentrations of gelatin/mTG solution were loaded into the dishes with 2 ml for each dish, and the dishes were incubated at 37 °C for 2 h. After gelling, the hydrogel-containing dishes were weighed again; 2 ml of PBS was added into each dish; and the dishes were cultured at 37 °C with 5% CO_2_ for eight weeks. PBS was changed every 3 days. At different time points, three dishes were removed for testing; PBS was removed completely; and the hydrogel-containing dishes were weighed. The remaining hydrogel mass was obtained by calculating the weight variation of each dish. Finally, the degree of degradation was expressed as a percentage of the remaining hydrogel mass versus the initial hydrogel mass. To prepare cell-containing hydrogels, 2 ml of gelatin/mTG solution mixed with 5.0 × 10^6^cells was added into a 35 mm culture dish. The rest of the steps were the same as the previous methodology, except that the culture medium was changed to cell expansion medium (see ‘Primary culture of ADSCs’). Cell culture medium was replaced every 3–4 days.

### Primary culture of ADSCs

Animal study was approved by the Institutional Animal Care and Use Committee (IACUC) of Sichuan University, all experiments were performed in accordance with the guidelines of IACUC of Sichuan University. Subcutaneous adipose tissue was obtained from a 150 g Sprague–Dawley rat. Harvested tissue was enzymatically dissociated using 1 mg/ml collagenase type I (Sigma, MO, USA) in high-glucose Dulbecco’s modified Eagle’s medium (DMEM; Hyclone, UT, USA). Digestion was carried out under continuous agitation for 45 min at 37 °C and followed by centrifugation at 283 g for 7 min. The pelleted cells were then harvested and plated on 25 mm^2^ cell flasks. The initial plates were denoted by Passage 0 (P0). At 24 h intervals, cultures were washed with PBS to remove contaminating erythrocytes and other non-attached cells. This procedure was repeated every 24 h for three days. The plating medium consisted of high-glucose DMEM, 15% fetal bovine serum (FBS; Invitrogen, CA, USA), and 1% penicillin/streptomycin (P/S; Hyclone, UT, USA). Cells were maintained at 37 °C with 5% CO_2_ and fed two times per week. When cultures reached confluence within 5–7 days after the initial plating, the adherent cells were detached with 0.25% trypsin/EDTA and then either replated at 5.0 × 10^4^ cells/cm^2^ or immediately used in experiments. Cultures were passaged every 3–5 days, and the culture medium was replaced by expansion medium, which consists of high-glucose DMEM, 10% FBS, and 1% P/S.

### Cell viability in 2D culture

Gelatin/mTG solutions with various gelatin concentrations (2%, 4%, 6%, 8% and 10%) were prepared as described above: the solutions were added into six-well tissue culture plates (TCPs), with 2 ml placed in each well. The solutions were incubated at 37 °C for gelling for 2 h. After washing thrice with sterile PBS, the hydrogels were ready for cell 2D seeding. ADSCs were digested and centrifuged at 283 g, and the supernatant was discarded. ADSCs were then seeded on the hydrogel surface at 1.0 × 10^5^ cells per well. Cells were cultured in the expansion medium and observed daily under an inverted optical microscope (CKX41, Olympus, Tokyo, Japan). After 14 days of cultivation, live/dead staining assay was employed to assess the viability of cell populations on 2D hydrogel substrates. Samples were washed thrice in sterile PBS and incubated at 37 °C for 30 min in a solution containing 2 µM calcein—AM (Sigma, St. Louis, MO, USA) and 2 µM propidium iodide (PI; Sigma, St. Louis, MO, USA) in PBS. After incubation, samples were washed again and pictures were obtained by using an inverted fluorescent microscope (XDS30; Ningbo Sunny Instruments Co., Ltd., Zhejiang, China) equipped with a video camera (MD50; Mingmei, China).

### Cell viability in 3D culture

ADSCs were prepared as described above and cell density was adjusted to 5.0 × 10^6^cells/ml. Cell suspensions were mixed with different concentrations (4%, 6%, and 8%) of gelatin/mTG solution at 1:9 volume ratio. Aliquots of 100 µl were loaded into six-well TCPs and incubated at 37 °C for 2 h. After gelling, 2 ml of culture medium was added in each well and the TCPs were incubated at 37 °C in 5% CO_2_. The medium was changed every three days. Cell morphology was monitored daily under an inverted optical microscope. After two weeks of cultivation, cell viability was tested via the calcein—AM/PI assay as mentioned before, and images were captured using a confocal laser scanning microscope (A1si, Nikon, Japan).

### Cell proliferation assay

ADSCs were seeded on the surface of 2–8% hydrogels (2D culture) or embedded into hydrogels (3D culture) as described above at 5.0 × 10^4^ cells per well of 24-well TCPs. Cells were cultured in expansion medium for four weeks, and medium was changed every 3 days. At each time point, cells/hydrogel constructs from three parallel samples were rinsed thrice with PBS and incubated in 100 µl of 10% Alamar Blue solution (Yeasen, China) for 3 h. Subsequently, the incubation solutions were transferred to a 96-well TCP, and fluorescence was measured with a plate reader using excitation/emission wavelengths of 530/590 nm. ADSCs seeded on TCPs served as a negative control.

### Cardiac differentiation of ADSCs

To investigate the differentiation status of encapsulated stem cells, ADSCs from the third passage were encapsulated in 6% gelatin/mTG hydrogels with the method described above (‘Cell viability in 3D culture’). After forming hydrogels, encapsulated cells were divided into two groups: one group was treated with normal cell expansion medium (see ‘Primary culture of ADSCs’), and the other was treated with differentiation medium. The differentiation medium consisted of DMEM with 1% P/S supplemented with 2% horse serum (HS; Hyclone, UT, USA) and 50 µg/ml *L*-ascorbic acid. The medium was changed twice a week, and a 14-day culture was performed. Control groups were seeded on TCPs supplemented with cell expansion medium or with differentiation medium for the same period.

The expression of cardiac-specific genes NK2 homeobox 5 (Nkx2.5), alpha cardiac myosin heavy chain 6 (Myh6), gap junction protein alpha 1 (Gja1), and myocyte enhancer factor 2C (Mef2c) in hydrogel-encapsulated cells and in control groups were measured by real-time reverse transcription-polymerase chain reaction (RT-PCR). Total RNA was extracted using Trizol reagent (Life Technologies). Complementary DNA was synthesized from 1 mg total RNA by employing RevertAid First Strand cDNA Synthesis Kit (K1622; Thermo Scientific, Vilnius, Lithuania). Complementary DNA samples were subjected to PCR amplification using Luminaris Color HiGreen qPCR Master Mix (K0391; Thermo Scientific, Vilnius, Lithuania). The DNA sequences of primers are listed in Table 1. PCR was performed with a real-time PCR Detection System (iCycler IQ5; Bio-Rad, Hercules, CA, USA). Cycles were programmed as follows: 95 °C for 10 min, 40 cycles of 15 s denaturation at 95 °C, 30 s at an annealing temperature of 57 °C, 30 s extension at 72 °C, with a final extension at 72 °C for 10 min. The 2^−ΔΔ*Ct*^ method was used to evaluate relative mRNA expression levels for each target gene. The expression of the housekeeping gene *β*-actin was employed for internal normalization. The product size was confirmed by running 10 µl of samples on 2% agarose gel electrophoresis.

**Table 1:** Sequences of PCR primers.

Gene	Primer sequence (5′-3′)	Accession no.	Product (bp)
Nkx2.5	F: CCTCGGGCGGATAAGAAAG R: ACTTGTAGCGGCGGTTCT	NM_053651.1	262
Myh6	F: ACACCAGCCTCATCAACCA R: CCTTCTCCTCTGCGTTCCT	NM_017239.2	105
Gja1	F: TGTGATGAGGAAGGAAGAGAAG R: TTGAAGAGGATGCTGATGATGT	NM_012567.2	192
Mef2c	F: CGGACTGATGAAGAAGGCTTAT R: GGCTGTGACCTACTGAATCG	XM_003749164.1	254
*β*-actin	F: GGACCTGACAGACTACCTCAT R: GAACCGCTCATTGCCGATA	NM_031144.3	217

### Cell migration study in a 3D culture model

The migration ability of ADSCs in 3D hydrogels was evaluated by using a specially designed model in which cells were seeded. In brief, 2–8% gelatin/mTG solutions were loaded in six-well TCPs at 2 ml volume in each well and incubated at 37 °C for 3 h. Subsequently, a 2 mm radius hole was made at the center of each hydrogel by using a punch, and 25 µl of 8% gelatin/mTG solution mixed with 1.0 × 10^7^ cells was filled into the hole. The added gelatin/mTG/cells constructs were set to solidify for 2 h and then each well was supplemented with 2 ml expansion medium. The cells were cultured at 37 °C with 5% CO_2_ for 14 days, and the culture medium was changed every 3 days. Meanwhile, the gelatin/mTG/cells constructs were monitored daily to observe the cell migration from the transplanted site into the surrounding matrix. Cell migration images were captured, and cell migration distances from the hole edge to the cell outgoing front in all directions were measured via image analysis software (Image Pro Plus 6.0; Media Cybernetics, Rockville, MD, USA). Afterward, the average migration distance was calculated for statistical analysis.

### *In vivo* implantation of hydrogel

The *in vivo* biological response to gelatin/mTG hydrogels was assessed at four weeks in subcutaneous SD rat models (age 6–7 weeks) with a total of four rats included in this study. Hydrogels with 4% and 8% gelatin were prepared as described above, and the hydrogels were cut into sheets of 10 mm × 10 mm × 1.5 mm size. Prior to surgery, the rats were anesthetized with 10% chloral hydrate and the areas of surgery were shaved and disinfected with iodophors. A pair of hydrogel sheets with 4% and 8% gel concentrations was surgically placed within subcutaneous pockets located on both sides of an adult rat dorsum. Three rats were implanted with hydrogels, and the last rat was subjected to surgery, but no hydrogel was implanted. The incision was covered in Betadine ointment after surgical suture and assessed for signs of infection for three days after surgery. All rats survived the surgical procedure without surgical complications. After four weeks the rats were sacrificed and the implants were harvested and sectioned at 10 µm thick with a cryosection microtome (Leica CM1950; Leica, Wetzler, Germany). The sections were fixed in 4% paraformaldehyde and processed for histological analysis using hematoxylin/eosin staining.

### Statistical analysis

Data are presented as mean ± SD. Statistical analyses were performed using SPSS software (version 14.0). Statistical significance between two groups was determined by Student’s *t*-test. Results for more than two groups were evaluated by one-way ANOVA with least-significant difference (LSD) test. *P* < 0.05 was considered statistically significant.

## Results

### Evaluation of gelation time and gel strength

**Figure 1:**
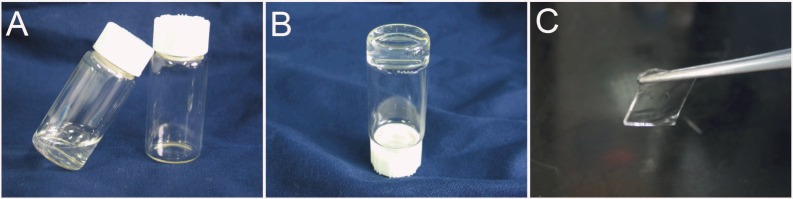
Morphology of 4% gelatin/mTG hydrogel. (A) Gelatin/mTG mixing solution. (B) The gelatin/mTG solution turned to a hydrogel at 37°C and the gel state was evaluated by bottle-invert method. (C) With sufficient gel strength, a piece of 4% gelatin/mTG hydrogel can be held by a pair of forceps.

Gelation time of the gelatin/mTG solution was evaluated at 37 °C via a bottle-invert method (Fig. 1). Depending on mTG dosage or gelatin percentage in gelatin/mTG solution, the gelation time was verified to be controllable. Decrease in either mTG dosage or gelatin percentage extended gelation time. When mTG dosage was kept constant at 10 U/g pro, the gelation time was inversely proportional to gelatin concentration (shown as Table 2). The 10% gelatin/mTG solution only took 15 s before arriving gel state. By contrast, the 1% gelatin/mTG solution retained a flow state and did not become a hydrogel.

**Table 2:** Gelation time and gel strength determined by gelatin percentage (mTG dosage = 10 U/g pro).

Gelatin percentage	1%	2%	3%	4%	5%	6%	8%	10%
Gelation time (min)	–	124.33 ± 6.28^∧^	89.5 ± 2.59^∧^	50.33 ± 4.27^∧^	38.83 ± 0.98^∧^	25.5 + 0.55^∧^	20.5 ± 0.55^∧^	15.67 ± 0.52^∧^
Gel strength (kPa)	–	–	2.80 ± 0.28^#^	5.49 ± 0.31^∗^	12.42 ± 0.83^#∗^	20.94 ± 1.48^#∗^	34.99 ± 1.49^#∗^	54.59 ± 5.12^#∗^

**Notes.**

^∧^ , ^#^, ^∗^ *P* < 0.01, when compared to each other.

To investigate the mechanical properties of gelatin/mTG hydrogels, we employed a digital pull and push dynamometer to evaluate gel strength. We found that gel strength is proportional to the concentration of gelatin solution. For example, gel strength increased rapidly as gelatin concentration increased from 2% to 6% and retained a relative constant strength thereafter even if increasing gelatin concentration to 10%. On the other hand, considering that gel strength may also be correlated with mTG dosage we applied a variety of mTG dosages with 4% gelatin solution to test their gelation time and gel strength (shown as Table 3). The results showed 10 U/g pro could be a compromised dosage that could bring about adequate gel strength and minimize the side effects possibly caused by excessive mTG.

**Table 3:** Gelation time and gel strength determined by mTG dosage (Gelatin percentage = 4%).

mTG dosage (U/g pro)	2	5	10	20	40
Gelation time (min)	180.33 ± 8.02^$^	97.33 ± 4.27^$^	50.33 ± 4.27^$^	34 ± 1.41^$^	20.66 ± 0.82^$^
Gel strength (kPa)	1.65 ± 0.10^#∗∧^	3.55 ± 0.56^#∗∧^	5.49 ± 0.31^#^	5.50 ± 0.29^∗^	5.58 ± 0.34^∧^

**Notes.**

^$^, ^#^, ^∗^, ^∧^ *P* < 0.01, when compared to each other.

### Hydrogel degradation performance

**Figure 2:**
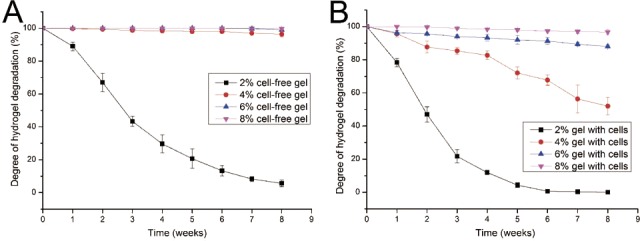
Degradation properties of gelatin/mTG hydrogels containing with or without cells. (A) Degradation curves of cell-free hydrogels. (B) Degradation curves of cell-containing hydrogels.

Given that cells seeded in a hydrogel may secrete several proteases, such as collagenase, which could lead to hydrogel degradation, the degradation performance of gelatin/mTG hydrogel should be evaluated. The degradation test was carried out in two parallel groups: cell-free hydrogels and cell-containing hydrogels. In the test of cell-free hydrogels (Fig. 2A), the degradation rate of 2% concentration of hydrogels was inversely proportional to culture time. Within three weeks, the hydrogels lost more than half of their original mass. At the eighth week, only approximately 5.6% of original mass remained. For the other three kinds of hydrogels (4%, 6% and 8%), their degradation curves were different. Apparent degradation in these hydrogels was not observed until the eighth week. For the hydrogels with 4% concentration gelatin, 3.6% mass loss was observed

In the test of cell-containing hydrogels (Fig. 2B), we found that 2% hydrogels degraded more rapidly than cell-free hydrogels. For three weeks, nearly 80% of hydrogel mass was lost. Moreover, the 4% hydrogel also showed significant degradation. At the eighth week, nearly half of the hydrogel mass was lost. However, the 6% and 8% hydrogels did not show severe degradation. For 6% hydrogel, approximately 12% of gel mass was lost after eight weeks of incubation; meanwhile for 8% hydrogels, mass loss was less than 4%.

### Cell morphological observation from 2D culture

**Figure 3:**
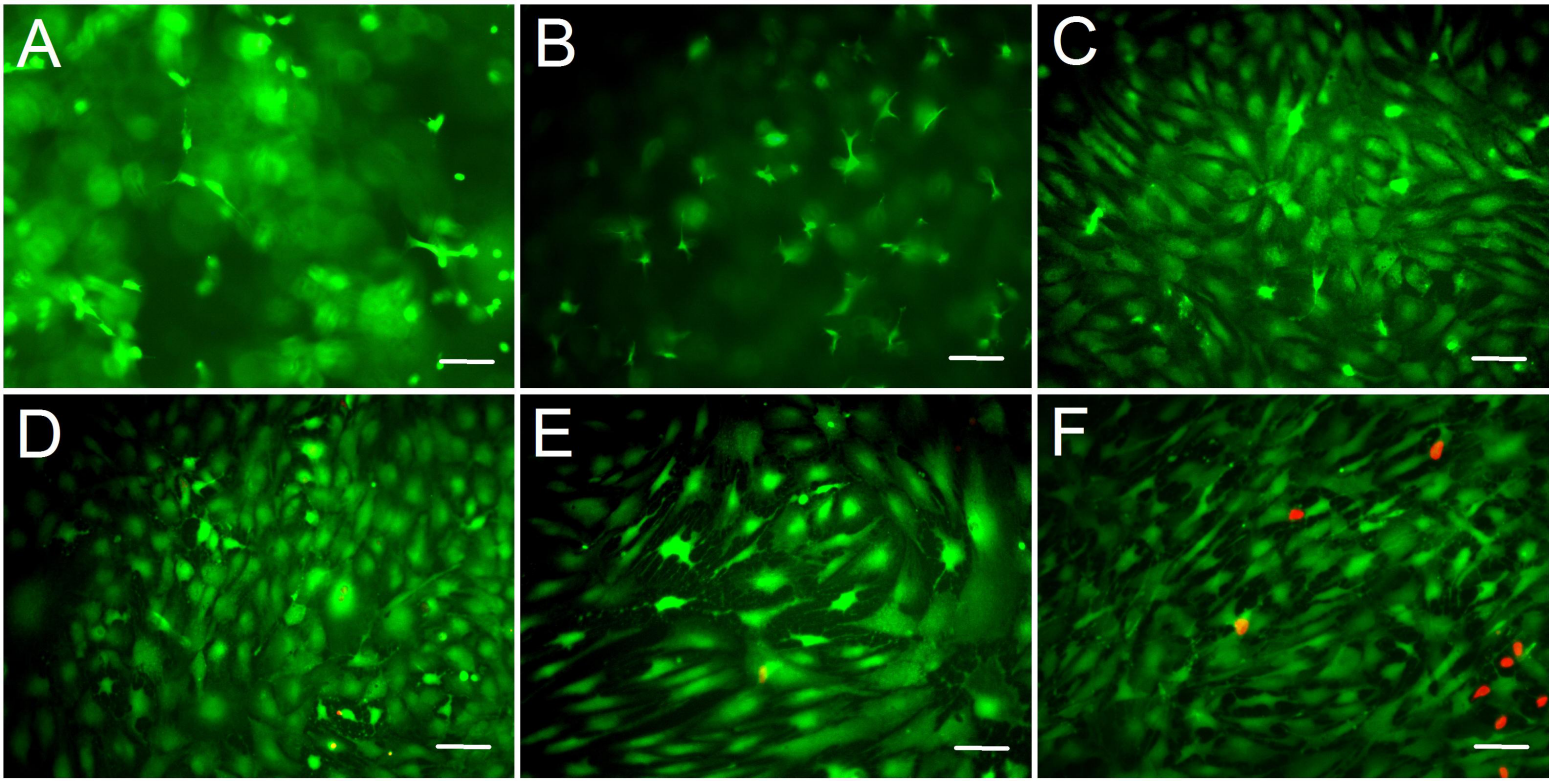
Cell live/dead fluorescent staining of ADSCs seeded on the surface of gelatin/mTG hydrogels. ADSCs cultured on (A) 2%, (B) 4%, (C) 6%, (D) 8%, and (E) 10% gelatin hydrogel and on (F) TCP at day 14 after seeding. Living cells were stained by calcium—AM (Green), and dead cells were stained by PI (Red). Scale bars = 100 µm.

We assessed the biocompatibility of gelatin/mTG hydrogels and its effect on cell adhesion. ADSCs were seeded on the surfaces of various hydrogels for two weeks. Cell viability was evaluated via live/dead staining assay. Cell staining showed that the vast majority of ADSCs cultured on 2%–10% gels were stained green (on behalf of living cells), and only few cells were stained red (on behalf of dead cells). This finding means that these hydrogels present good biocompatibility and are suitable for cell 2D culture. In addition, regarding cell morphology, the effects of different concentrations of hydrogel materials on cell growth and adhesion behavior were different. The cells seeded on 2% hydrogels plunged into hydrogels and grew inside owing to weaker gel strength, which led to more rounded or stick-shape cells appearing than other hydrogels (Fig. 3A). As the hydrogel concentration increased to 4%, cell shape progressively assumed more of barbed-like or caltrop-like patterns, which represented the cell pseudopodia stretching out in different directions in a 3D gel space (Fig. 3B). With further increasing hydrogel concentration (Figs. 3C and 3D), cells gradually spread out and grew in size. When the hydrogel concentration increased to 10% (Fig. 3E), the cell growth pattern on the gel was similar to that on TCP (Fig. 3F).

### Cell morphological observation from 3D culture

**Figure 4:**
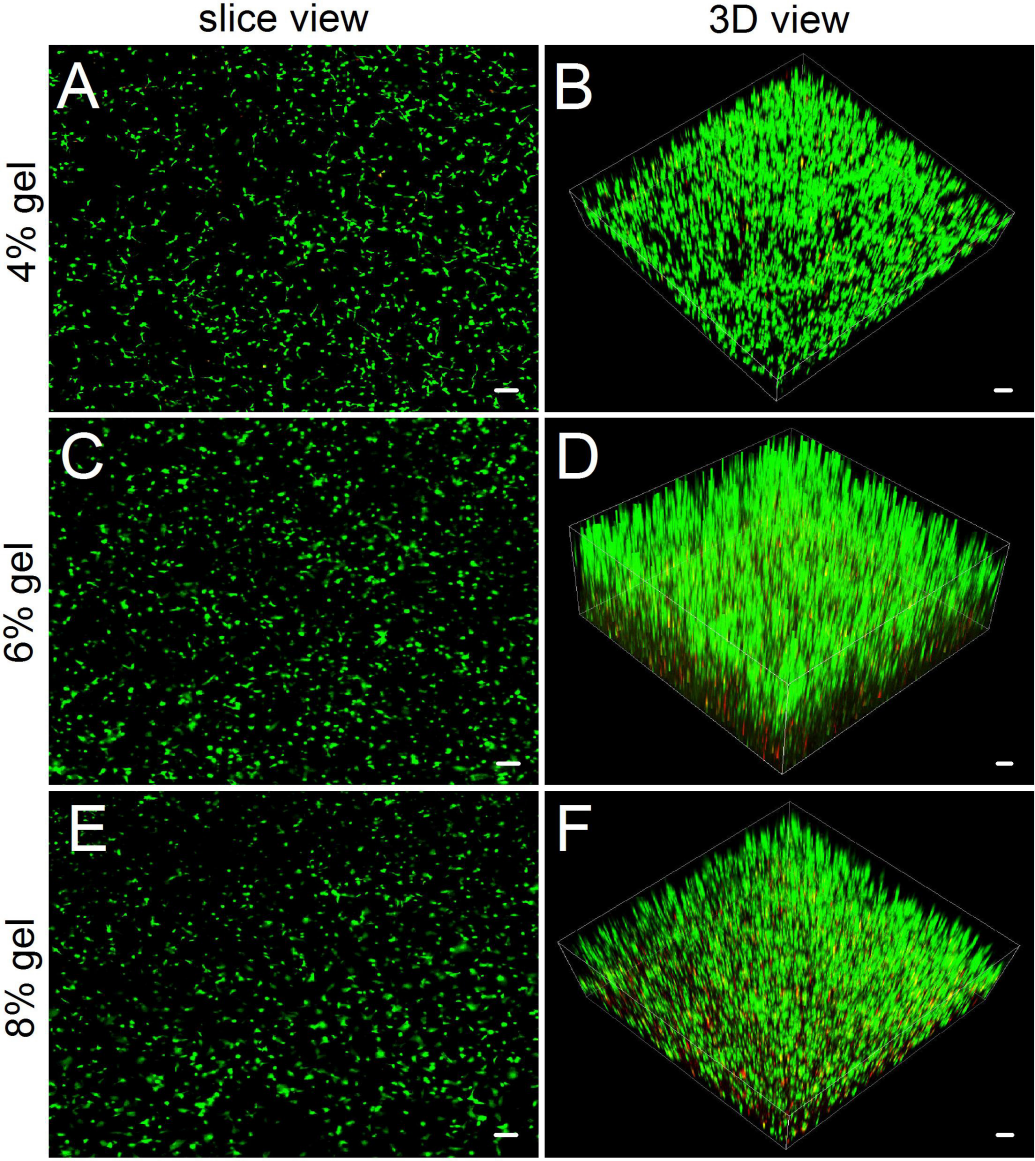
Observing cell viability in 3D cultures by a confocal laser scanning microscope. (A, C, E) In slice views, ADSCs embedded into the 4–8% concentrations of gelatin/mTG hydrogel exhibited good viability with abundant protrusions. (B) In 3D views, ADSCs are distributed in hydrogels extensively and exhibit vigorous proliferation in 3D culture. Scale bars = 100 µm.

Compared with hydrogel-surface culture, the 3D culture of embedding ADSCs into a hydrogel better mimicked *in vivo* cell growth. Three concentrations (4%, 6% and 8%) of hydrogels were included in 3D culture experiments. As for 2% hydrogels, owing to their relatively low gel strength and susceptibility to degradation, we did not consider the hydrogels as experimental candidates. As for 10% hydrogels, we also excluded them from the experiment because in both the hydrogel degradation test and the 2D cell culture experiment, their performances did not show apparent difference with that of 8% hydrogels. In 3D culture, we found that the encapsulated ADSCs from all tested hydrogels present good viability (Fig. 4). After two weeks of culture, the slice views photographed by laser scanning confocal microscopy showed that the cells distributed evenly on the hydrogels with numerous cell protrusions. In the synthesized 3D images, we clearly saw that the ADSCs almost filled all areas inside the hydrogels. However, with the increase in hydrogel concentrations, some of the cells residing deep in these hydrogels died because of insufficient nutrient metabolism caused by the compact hydrogel.

### Evaluation of cell proliferation in 2D and 3D culture

**Figure 5:**
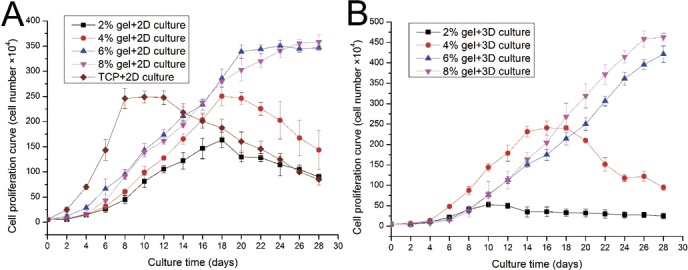
Cell proliferation capabilities assessed by Alamar Blue assay after four weeks of 2D or 3D culture. (A) Growth curves of ADSCs cultured on the surface of different hydrogels; (B) growth curves of ADSCs embedded into different hydrogels.

ADSC proliferation capabilities were assessed by Alamar Blue assay. During four weeks of 2D culture, significant difference in cell growth behavior was observed (Fig. 5A). On day 2, evident differences were not observed among the cells cultured on different substrates. However, from day 4 to day 12, the cells on TCP apparently showed a higher proliferation rate compared with cells cultured on the gel surface. However, after 12 days of culture, the cell number on TCP almost linearly decreased as the cells stopped growth caused by cell contact inhibition, and some cells were detached from the TCP surface. From the 2D culture experiments we found cell proliferation ability was almost proportional to the concentration of hydrogel. We also found that cell growth on lower hydrogel concentration (2% and 4%) decreases on day 18 possibly because of degradation of these materials, which led to the loss of the corresponding mechanical support. For 8% or 10% hydrogels, the cell number remained increasing despite the cell growth rate started to slow down after three weeks of culture.

ADSC proliferation in 3D culture was carried out using Alamar Blue assay (Fig. 5B). Cellular proliferation behavior in 3D culture presented a similar situation as that in 2D culture. The hydrogels at 8% or 10% concentration shows good performance in supporting cell growth because the cell number in these hydrogels continued to increase even after four weeks of culture. Cells on hydrogels with 4% gelatin concentration exhibit fast growth rate in the first two weeks; however, cells begin to fall down from day 18 after cell seeding. The 2% hydrogels exhibit poor performance and few cells survived in hydrogels after two weeks of culture. We speculate that the 2% hydrogels present weaker mechanical properties, together with the degradation of collagenase secreted by the residing cells, leading to poor performance in supporting cell 3D growth.

### Relative mRNA expression of encapsulated ADSCs

**Figure 6:**
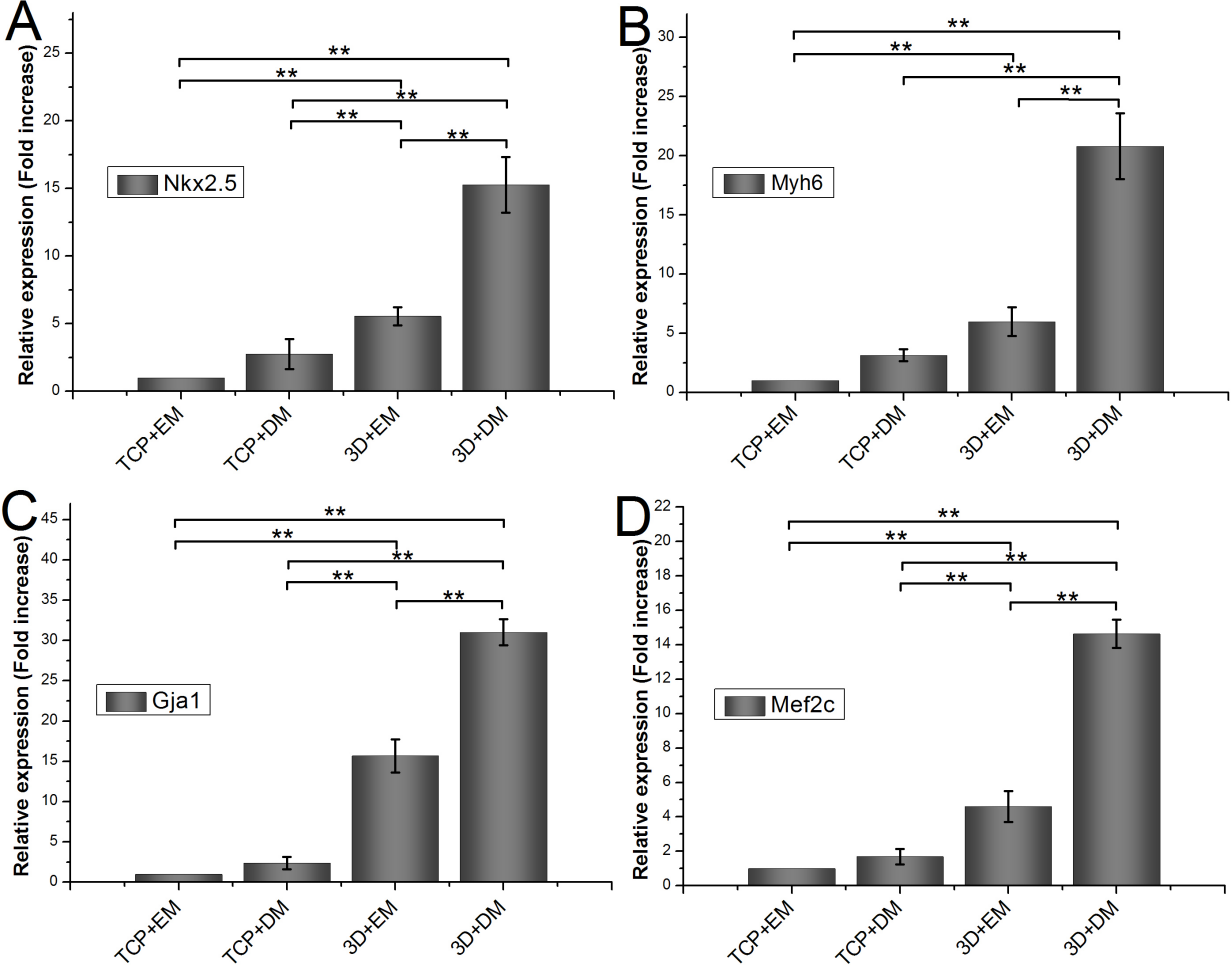
Cardiogenic gene expression of encapsulated ADSCs in cell expansion medium (3D+EM) or differentiation medium (3D+DM) for two weeks; ADSCs cultured on TCPs in expansion medium (TCP+EM) or differentiation medium (TCP+DM) were also evaluated. The expression levels of cardiac markers (A) Nkx2.5, (B) Myh6, (C) Gja1, and (D) Mef2c were detected by qPCR. All values are normalized to the expression level of *β*-actin. (**, *p* < 0.01).

To measure the effects of gelatin/mTG hydrogel on cardiogenic differentiation of ADSCs, we evaluated cardiac markers Nkx2.5, Myh6, Gja1, and Mef2c were evaluated by real-time quantitative PCR. All values are normalized to the expression level of *β*-actin. The mRNA expression level of ADSCs cultured on TCPs in cell expansion medium (negative control) is indicated as “1.” Each measure of gene expression in the encapsulated ADSCs is presented as a fold change over that of negative control. The results are shown in Fig. 6. The encapsulated ADSCs in differentiation medium present the highest mRNA expression level among all detected samples; their expression levels of Nkx2.5, Myh6, Gja1, and Mef2c are 15.26 ± 2.06-, 20.80 ± 2.79-, 31.01 ± 1.62-, and 14.64 ± 0.82-fold, respectively. For the encapsulated ADSCs in normal cell expansion medium, their mRNA expression levels of Nkx2.5, Myh6, Gja1, and Mef2c are 5.56 ± 0.67-, 5.97 ± 1.22-, 15.69 ± 2.05-, and 4.60 ± 0.91-fold higher compared with negative controls, respectively. No statistical difference was found in cells cultured on TCPs in the differentiation medium compared with negative controls.

### Evaluation of cell migration in 3D hydrogels

**Figure 7:**
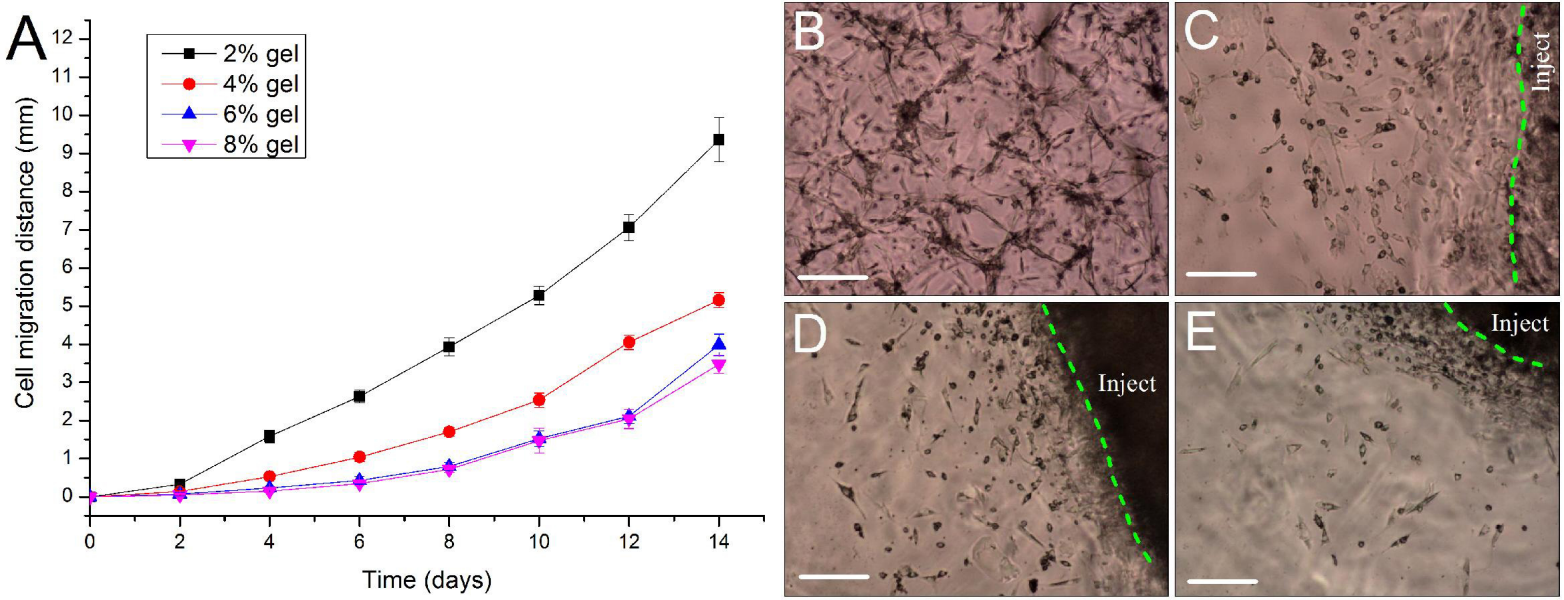
Evaluation of cell migration in 3D hydrogels. (A) Migration distance of the released ADSCs crawled outward from the edge of the *in vitro* implantation site into the surrounding hydrogels of various hydrogel concentrations. (B–E) At day 10 after implantation, the cells were released from the injected gelatin/mTG hydrogel and crawled into the surrounding hydrogels at concentrations of (B) 2%, (C) 4%, (D) 6%, and (E) 8%. “Inject” denotes the injected gel; the green dash line denotes the edge of the injected gel. Scale bars = 200 µm.

For repairing the damaged tissue and organ, cells/scaffold construct implanted into the body should be able to release cells from the scaffold into the surrounding tissue. In this study, we designed a 3D cell migration model to simulate the *in vivo* transplant environment. The experiments show that cell migration activities started as early as the 2nd day after *in vitro* hydrogel implantation and continued to the end of the experiments. Low concentration of hydrogels favors cell migration. With the increase in gel concentration, the hydrogels became dense, and the average migration distances of ADSCs encapsulated in the hydrogels were decreased. After two weeks cells in the 2% hydrogels migrated by 9.36 ± 0.58 mm with an average migration speed of about 0.67 mm/day. By contrast, in the other three hydrogels cells migrated by about 3–5 mm in the same period (Fig. 7A). The experiments also showed that the released cells from the cells/hydrogel construct maintained an extremely strong vitality and proliferative capacity during two weeks of experiments (Figs. 7B–7E).

### *In vivo* response to gelatin/mTG hydrogels

**Figure 8:**
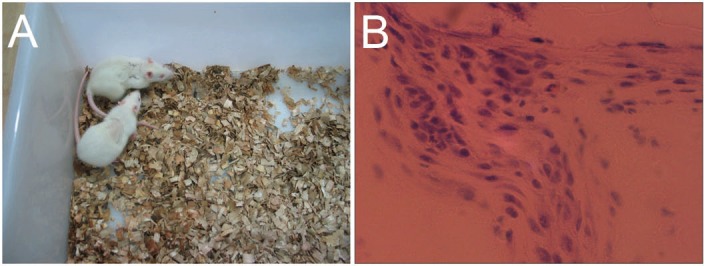
*In vivo* biological response to the gelatin/mTG hydrogels was assessed in subcutaneous SD rat models. (A) Wounds of the rats were healed without the formation of scar tissue in a week. (B) A small number of cells had invaded into the samples with 4% hydrogel, as detected by hematoxylin and eosin staining when implants were harvested after four weeks of experiments. Scale bars = 100 µm.

To characterize the local tissue response to gelatin/mTG materials, we implanted 4% and 8% concentration hydrogels subcutaneously into an adult SD rat model and explanted after four weeks for analysis. Approximately 6–8 h after surgery, the rats could eat and drink, and no breathing difficulties and unusual activities were observed. Their wounds were healed without the formation of scar tissue within a week (Fig. 8A). The 8% hydrogels could be clearly observed under the skin throughout the experiment period, indicating that the hydrogels are not degraded significantly. However, the 4% hydrogels could only be observed in the first two weeks and are difficult to identify after another two weeks. We speculated that material degradation had occurred. After four weeks, the rats were sacrificed, and the implants were harvested, thus confirming our speculation. All 4% concentration samples were severely eroded, but no macroscopic signs of inflammation or toxicity were evident in the tissue surrounding the implants. As for 8% hydrogel samples, apparent evidence of capsule formation was found around the implants, and only slight degradation occurred. Hematoxylin and eosin staining of sections throughout the explanted hydrogels showed no apparent infiltration of cells into the 8% hydrogels, but a small number of cells had invaded into the 4% hydrogel samples (Fig. 8B). We can see from morphological observation, that the cells were not macrophages or inflammatory cells but may be endothelial cells instead, migrating from surrounding tissues, to form capillary networks inside hydrogels.

## Discussion

Evaluation of the performance of a biological scaffold usually involves two important aspects: whether the hydrogel can support cell adhesion and cell proliferation and determining whether the mechanical strength of the hydrogel material can meet the requirements of practical applications. For example, collagen-based hydrogel exhibits excellent cell adhesion property but poor mechanical performance. Synthetic hydrogel materials, such as polyethylene glycol and poly lactic acid, exhibit excellent mechanical properties, but the lack of cell binding domains leads to weak cell adhesive ability. Moreover, the degradation rate of most synthetic materials *in vivo* is slow, thereby affecting tissue regeneration. One of the advantages of choosing gelatin as a biological scaffold is its abundance of cell binding domains. Gelatin is a common structural protein and is obtained from the degraded product of collagen protein, but the majority of the amino acid sequences of collagen is retained. In particular, the molecular weight of gelatin is smaller than that of collagen, and the space conformation of the gelatin protein is simpler, thus exposing more cell binding sequences of Arg–Gly–Asp (RGD) that can be identified by cellular integrin proteins. Therefore, gelatin hydrogel possesses excellent cell-response features, which can support cell proliferation, migration, differentiation, and development into a complex organizational structure.

Given that the mechanical properties of the non-crosslinked gelatin solution are extremely weak, gelatin cannot be directly used as a biological scaffold material. Gelatin is often used as substrate material in cell culture to promote cell adhesion on TCP. Gelatin can also be used as a part of composite material to increase cell biocompatibility (Han et al., 2015; Nieto-Suarez, Lopez-Quintela & Lazzari, 2016). Enzymatic crosslinking is a new approach in preparing gelatin hydrogel. Transglutaminases are a class of natural enzymes; there are at least eight genetically distinct transglutaminases in mammalian tissue, blood, ECM and on cell surfaces. All of these are Ca^2+^ dependent and are related to essential homeostatic processes such as blood coagulation (Factor XIIIa); cell differentiation, death and ECM stabilization (tissue transglutaminase); and maintenance of epidermal integrity (keratinocyte transglutaminase). Transglutaminases have a transamidation activity and catalyzes the acyl-transfer reaction between the ε-amino group of lysine and the *γ*-carboxyamide group of glutamine in proteins. mTG is Ca^2+^ independent and has transamidation ability similar to that of the mammalian versions (DM et al., 2006; Paguirigan & Beebe, 2007). The process of crosslinking gelatin/mTG is irreversible in physiological temperature range. The approach overcomes the shortage of reversibility in the obtained hydrogels via conventional physical crosslinking method. In addition, our experiments showed that the gelatin/mTG hydrogels possess high plasticity, and their mechanical properties are adjustable. These hydrogels can be used as preshaping hydrogels for cell inoculation or mixed with cells in solution and implanted *in vivo* by injection; subsequently, hydrogels are formed according to the spatial shape of the implantation site.

In our experiments, we found that the mTG is not cytotoxic, given that enzymatic crossslinking prevents potential toxicity caused by the residue of chemical crosslinkers. In the study of mTG dosage screening, we found that 2 U/g pro could crosslink 4% gelatin solution into a hydrogel; however, the process of crosslinking was time-consuming. Increasing mTG dosage could shorten the gelation time and could promote gel strength. A 10 U/g pro could be a proper dosage; a dosage higher than this does not significantly increase the gel strength. We also tried to use the dosage of 40 U/g pro to crosslink the gelatin hydrogel for cell culture; however, we did not found the significant differences in cell morphology and proliferation between the experiment group with 40 U/g pro dose and the control group with 10 U/g pro dose. Therefore, we concluded that a suitable mTG dosage is 10 U/g pro.

In evaluating the intrinsic properties of hydrogels, gelation time is a notable factor. For example, when mixing gelatin hydrogel with cells in 3D culture, we expect cells to be evenly suspended in the hydrogel. However, in the actual operation, if the gelation time is poorly controlled, a uniform cell distribution is difficult to achieve. Given that gelation time is related to the concentration of gelatin solution, the low-concentration gelatin solution exhibits long gelation time. When the gelatin/mTG solution was mixed with cells in the state prior to forming a gel, the cells will be precipitated by gravity, and the aim of uniform cell distribution cannot be achieved. High-concentration gelatin solution exhibits short gelation time. Therefore, the cells must be quickly mixed with the gelatin solution prior to gelling; otherwise, abundant bubbles will appear in the resultant hydrogel. With further stirring of the gel, the mechanical structure of the hydrogel will be undermined.

In this study, we found that hydrogel concentration affects cell growth morphology and cell proliferation. In 2% gelatin hydrogels, the cell growth morphology showed a round or stick-like shape because of the relatively low gel strength. Coupled with faster hydrogel degradation rate, low-concentration hydrogels cause significant cell loss in the cell proliferation experiments. Therefore, 2% gel was not used for cell migration and subcutaneous implantation. In 2D cell culture experiment, the high-concentration hydrogel exhibited a similar cell growth pattern to that on TCP. However, we found certain growth behavior differences between the cell cultured on hydrogel and on TCP: upon reaching confluence, the cells stopped growth and began detaching from the TCP culture surface. By contrast, cells on hydrogel maintained excellent adhesion owing to the abundant integrin binding domains provided by the gelatin hydrogel. In addition, the cells gradually grew into the hydrogel inside and spread out all over the gel space. This growth mode enhanced cell adhesion on the hydrogel surface, expanded cell growth space, and promoted cell proliferation. As seen from the 3D culture experiment results, the gelatin/mTG hydrogels exhibit excellent biocompatibility with extremely high cell survival rate. Moreover, the shape of encapsulated cells changed from a round shape to a barb-like shape, indicating that cell pseudopodia began stretching out and that cells were in a good growth status. Studies indicated that anchorage-dependent cells that remain round or in a non-adhesive state will eventually undergo cell apoptosis (Chen et al., 1997; Kuwahara et al., 2010).

ADSCs have attracted considerable attention because of their abundant stem cell sources. ADSCs compose a plastic-adherent cell population that includes vascular and adipocyte progenitor cells and adult multipotent mesenchymal stem cells (MSCs) (Madonna & De Caterina, 2010; Nguyen et al., 2016). In our previous study (Li et al., 2015), we examined the surface protein expression of rat ADSCs at Passage 3 via flow cytometry analysis. The results showed that CD29, CD44h, CD49d, and CD90 were positive in expression, whereas CD45 and CD106 were negative. Our results were in agreement with the reports in the literature (Bourin et al., 2013; Guo et al., 2016). In a non-differentiating medium, ADSCs can retain strong proliferation capability, maintain their phenotypes, and exhibit stronger multidirectional differentiation potential even after being passaged for 25 times (Zhu et al., 2008). ADSCs can differentiate into osteoblasts, chondrocytes, adipocytes, and cardiomyocytes via appropriate induction condition. Previous studies have indicated that the altering modulus of a hydrogel exerts a profound effect on the lineage commitment of stem cell (Chatterjee et al., 2010; Li et al., 2012). Li et al. (2012) encapsulated human MSCs in composited acrylamide hydrogels with three different moduli: 16, 45 and 65 kPa, after 14 days of *in vitro* culture, they found more than 76% of MSCs expressed cardiac specific markers in the 45 and 65 kPa hydrogels, and MSCs in the 65 kPa hydrogel had the highest differentiation efficiency. In our study, we evaluated the *in vitro* cardiac differentiation capacity of encapsulated ADSCs in 6% gelatin/mTG hydrogel (∼20.94 kPa) with or without cardiac induction medium. For the encapsulated ADSCs in normal cell expansion medium, the mRNA expression levels of Nkx2.5, Myh6, Gja1, and Mef2c were higher than those of cells cultured on TCPs. This result shows that the modulus of gelatin/mTG hydrogel can affect cell differentiation. Furthermore, the encapsulated ADSCs in differentiation medium presented higher levels of cardiogenic gene expression compared with those in the expansion medium, further confirming that the synergistic effect of the hydrogel modulus and differentiation medium can contribute to the cardiac differentiation of encapsulated ADSCs. By contrast, the differentiation medium alone could not induce ADSCs on TCPs toward cardiomyocyte fate.

In the cell migration experiments, we found that the cells were not only released from hydrogel encapsulation but also invaded the surrounding hydrogels. This finding shows that hydrogels are able to release entrapped cells and simultaneously accept the migrated cells from the surroundings. In short, cell release from the hydrogel encapsulation is controllable, and the cell migration rates can be regulated by changing the hydrogel concentration. However, increasing the hydrogel concentration will engender a certain degree of cell apoptosis in the hydrogel inside (under the hydrogel surface ∼200–300 µm). Therefore, if the thickness of cells/hydrogel construct exceeds 1 mm, we recommend that a multi-layered overlay should be considered for hydrogel design to facilitate the exchange of nutrients. When implanted into the body, the hydrogel can be invaded by the surrounding capillary network, thus solving the problem of blood supply to a certain extent. As we have seen in subcutaneous implantation experiments, some of endothelial-like cells had invaded the 4% hydrogel. If the experiment time is prolonged, capillary network may appear within the hydrogel. Moreover, the gelatin/mTG hydrogel can not only be used as a cell scaffold material, but also has a protective effect on the encapsulated cells against an inflammation response. Acute inflammatory response can produce unfavorable microenvironment for the injected stem cells. Inflammatory responses are time dependent, in the case of neural tissue, the most intense inflammatory response is within three days since stem cell transplantation (Okano, 2002; Kuwahara et al., 2010). From the experiment results of *in vivo* hydrogel implantation in our study, even the 4% hydrogel is able to maintain the gel state in the body for more than two weeks. This shows that gelatin/mTG hydrogel may serve as a cell barrier against acute inflammation response. Our next experiment will observe whether gelatin/mTG hydrogel can block inflammatory effects on the encapsulated cells.

In conclusion, the gelatin/mTG hydrogel is a potential scaffold material, either as a cell vehicle for *in vivo* implantation for wound healing, and soft and hard tissue repair, or as a drug delivery carrier for drug screening. This hydrogel can also be combined with other biological materials to form a composite material with more functions. We believe that this hydrogel material will be widely used in the field of tissue engineering and regenerative medicine as well as the field of drug release study. In our future study, the effect of different hydrogel moduli on ADSCs lineage commitment will be investigated, and immunofluorescence assay and flow cytometry analysis should be performed to further evaluate the cardiac differentiation status of encapsulated ADSCs. In addition, chromosomal karyotyping to ADSCs should also be examined for detecting chromosomal abnormality. Moreover, *in vivo* hydrogel/cells transplantation experiment will help us to better understand the function of gelatin/mTG hydrogel in tissue repair and organ reconstruction.

##  Supplemental Information

10.7717/peerj.2497/supp-1 Supplemental Information 1 Raw data for Table 2 Gelation time and gel strength determined by gelatin percentage (mTG dosage = 10 U/g pro)

10.7717/peerj.2497/supp-2 Supplemental Information 2 Raw data for Table 3

10.7717/peerj.2497/supp-3 Supplemental Information 3 Raw data for Figure 2

10.7717/peerj.2497/supp-4 Supplemental Information 4 Raw data for Figure 5

10.7717/peerj.2497/supp-5 Supplemental Information 5 Raw data for real time RT-PCR in Figure 6

10.7717/peerj.2497/supp-6 Supplemental Information 6 Raw data for Figure 7

## Additional Information and Declarations

### Data Availability

The following information was supplied regarding data availability: The raw data has been supplied as Supplemental Information.
